# Bridging Arrays and ADTs in Recursive Proofs

**DOI:** 10.1007/978-3-030-72013-1_2

**Published:** 2021-02-26

**Authors:** Grigory Fedyukovich, Gidon Ernst

**Affiliations:** 8 Eindhoven University of Technology, Eindhoven, The Netherlands; 9 Aalborg University, Aalborg East, Denmark; 10 Florida State University, Tallahassee, USA; 11 Ludwig-Maximilians-University, Munich, Germany

## Abstract

We present an approach to synthesize relational invariants to prove equivalences between object-oriented programs. The approach bridges the gap between recursive data types and arrays that serve to represent internal states. Our relational invariants are recursively-defined, and thus are valid for data structures of unbounded size. Based on introducing recursion into the proofs by observing and lifting the constraints from joint methods of the two objects, our approach is fully automatic and can be seen as an algorithm for solving Constrained Horn Clauses (CHC) of a specific sort. It has been implemented on top of the SMT-based CHC solver AdtChc and evaluated on a range of benchmarks.

### 1 Introduction

Relational verification is widely applicable during an iterative process of software development, when a high-level specification, a prototype implementation, or even an arbitrary previous version is compared to the current version and verified for the absence of newly introduced bugs. As software grows large, *compositionality* becomes a crucial factor to achieve scalability of relational verification tasks: reasoning about pairs of entire programs is reduced to reasoning about pairs of modules or isolated components of code. Proofs found for one component can be reused while reasoning about another component, or even the system in a whole. Successful examples in large-scale verification projects include a step-wise refinement in `seL4` [30] and the integration of model checking to software development workflow in `AWS C Common` [11].

In this work, we represent relational verification problems over *object-oriented programs* as Constrained Horn Clauses (CHC). A CHC is an implication in first-order logic that involves a set of unknown predicates. For a system of CHCs, we wish to find an interpretation for all predicates that validates all implications. CHCs are used in various tasks appearing in verification, e.g., finding loop invariants or function summaries. For relational verification, a system of CHCs can be constructed by pairing components of code of two versions in lockstep and supplying it with relational pre- and post-conditions [14,39,44,53]. State-of-the-art tools for solving CHC, e.g., [9,19,21,27,32], are based on Satisfiability Modulo Theories (SMT), e.g., [40,47], they gradually become more robust, as long as the programs under analysis do not have a *mixed use of data structures*.

Verification conditions of real-world problems involve data structures such as arrays and Algebraic Data Types (ADTs) of unknown size, expecting the proofs to capture (quantified or recursive) properties over countably infinite sets of elements. Arrays are being handled in loops and often require finding universally-quantified loop invariants [21]. ADTs, such as lists, maps, and sets, require reasoning by structural induction [47] and often rely on additional helper lemmas which are difficult to be synthesized automatically. For relational verification tasks, where one program is over arrays, and another is over ADTs, the solvers should likely reason over quantified formulas and induction *at the same time*, which is currently challenging for most of the automated tools.

We propose a set of new algorithms for solving CHCs constructed by pairing programs over arrays and ADTs. Because we deal with object-oriented programs, the data structures might be accessed and modified in any given method, and our pairing is done for each method separately. Relational proofs are synthesized over the data structures – they describe a relation that holds while *simultaneously traversing pairs of elements* by any of the methods. Our key idea is that not all methods may be needed for the actual synthesis. In fact, our algorithm generates a candidate proof by bridging a single pair of methods and then validates/repairs it on all others. In essence, we observe how pairs of inputs (or pairs of outputs) change the states, guess a candidate relation between elements of states, and (dis-)prove it on all other methods using an SMT-based theorem prover.

Our synthesis strategy is customized for different classes of benchmarks via so called *recipes*. We present two recipes for the list ADT that are applicable, respectively, for (1) stacks and queues, and (2) sets, multisets, and maps. They both discover nontrivial invariants that need a *recursive interpretation*. We independently generate its base and recursive cases. The key point in determining the relations is to automatically investigate how an input or an output affects the state. Finally, we discover auxiliary lemmas that provide additional properties about objects in isolation and help proving the inferred invariants are valid.

Importantly, in contrast to a more lightweight CHC setting over numerical theories (and even arrays) that can rely on an SMT solver to validate its recursion-free solutions, the validation of our *recursive solutions* is conducted by structural induction. We thus rely on recent advances in SMT-based *fully automated* theorem proving [55] that (since recently) supports arrays. The experiments have shown that the approach is reasonably fast in practice. Our contribution, while presented in the CHC context, can be lifted on the program analysis context and implemented in a range of robust verification tools that are designed to support compositionality [7,24].

The rest of the paper is structured as follows. A short outline on background and notation is given in Sect. 2. In Sect. 3, we give an overview of the approach. Then, Sect. 4 and Sect. 5 present our recipes. Finally, we give the evaluation details in Sect. 6, related work in Sect. 7, and conclude the paper in Sect. 8.

### 2 Preliminaries

An *object* $O = (St, Init, (Op_n)_{n \in [1,N]})$ is defined over internal states $St$, with initialization $Init(s)$ denoting initial states $s$, and methods $Op_n$, also called operations, for some identifier $n$ (which for simplicity is treated as a natural number in some finite interval, but later sections liberally refer to $Op_n$ by their name). Each operation $Op_n(in, s, s', out)$ defines transitions between a pair of states $s$ and $s'$ for a given input $in$, producing an output $out$. Moreover, each operation has an associated precondition $pre_n(in, s)$, ranging over the input and pre-state.

In this paper, we take a syntactic approach by representing states as tuples of variables. Specifically, we assume that $Init(s)$ and each operation $Op(in, s, s', out)$ is given as a predicate, i.e., as a characteristic formula, over the specified parameters, that holds for initial states, respectively, when the program can take a particular transition. Such a formula can be obtained from the source code by symbolic execution, and we assume that effect of loops inside operations is captured by quantified formulas, creation of which is an orthogonal problem. Hence, our approach is language agnostic.

We assume that the programs under consideration are deterministic, and we assume that $pre(in, s) \implies \exists s', out.\ Op_n(in, s, s', out)$. Note that for deterministic programs, the existential quantifier in $\exists s', out.\ Op_n(in, s, s', out)$ can be eliminated if $pre(in, s)$ holds as $s', out$ are functionally determined by $in, s$.

We aim at solving a *relational verification problem* over two objects and reduce it to *inductive invariant* inference over a *composition* of two objects.

**Definition 1.** *Two objects $A$ and $C$ are* equivalent *if there exists an inductive invariant $\boldsymbol{R}$ over a* composition *of these objects, which satisfies all clauses below. It connects two states $St^A$ and $St^C$ before and after each pair of operations $(Op_n^A, Op_n^C)_{n \in [1,N]}$.*

$$
\begin{cases}
\textbf{initialization:} & \\
\quad Init^A(as) \land Init^C(cs) \implies \boldsymbol{R}(as, cs) & \\
\textbf{consecution:} & \\
\quad \boldsymbol{R}(as, cs) \land Op_1^A(in, as, as', out^A) \land Op_1^C(in, cs, cs', out^C) \implies \boldsymbol{R}(as', cs') & \\
\quad \dots & \\
\quad \boldsymbol{R}(as, cs) \land Op_N^A(in, as, as', out^A) \land Op_N^C(in, cs, cs', out^C) \implies \boldsymbol{R}(as', cs') & \\
\textbf{safety: applicability:} & \\
\quad \boldsymbol{R}(as, cs) \land pre_1^A(in, as) \implies pre_1^C(in, cs) & \\
\quad \boldsymbol{R}(as, cs) \land pre_1^C(in, as) \implies pre_1^A(in, cs) & \\
\quad \dots & \\
\quad \boldsymbol{R}(as, cs) \land pre_N^A(in, as) \implies pre_N^C(in, cs) & \\
\quad \boldsymbol{R}(as, cs) \land pre_N^C(in, as) \implies pre_N^A(in, cs) & \\
\textbf{safety: outputs:} & \\
\quad \boldsymbol{R}(as, cs) \land Op_1^A(in, as, as', out^A) \land Op_1^C(in, cs, cs', out^C) \implies out^A = out^C & \\
\quad \dots & \\
\quad \boldsymbol{R}(as, cs) \land Op_N^A(in, as, as', out^A) \land Op_N^C(in, cs, cs', out^C) \implies out^A = out^C &
\end{cases}
$$

Implications in Def. 1 define a set of Constrained Horn Clauses (CHC) over an uninterpreted relation symbol $\boldsymbol{R}$. There are three types of constraints: (1) initialization, (2) consecution, and (3) safety. The third, safety, reflects the actual relational specification, i.e., the correspondence between the programs under analysis, in terms of the user-visible variables, namely the input $in$, and the respective outputs, $out$ and $out'$. Here, safety is divided into applicability (coincidence of preconditions) and equivalence of outputs, which together ensure that the two programs are observationally equivalent. To prove that this equivalence holds, one needs to infer a more complicated invariant $\boldsymbol{R}$ over the internal state. For this reason, we need the initiation and the consecution constraints: whatever happens due to each operation, the invariant is maintained, and by safety, the programs remain observationally equivalent indefinitely.

**Problem Statement:** We seek an interpretation of $\boldsymbol{R}$ that satisfies all constraints in Def. 1 simultaneously. This conventional formulation of a CHC task lets us to use any off-the-shelf CHC solver. However, the problem is undecidable in general, thus no solver guarantees to handle our specific tasks. Furthermore, existing solvers mainly support the lightweight arithmetic theories, and a few exceptions support also ADTs [27] and arrays [21,32]. To the best of our knowledge, there is no CHC solver that supports ADTs and arrays *at the same time*, and there is no CHC solver that synthesizes recursive solutions.

**Context:** The system of CHCs ensures that $A$ and $C$ can be substituted interchangeably in any calling context, and it is applicable to a wide range of techniques for formal program development. The focus on equivalence instead of subsumption is not essential for our work, and the presented approach works for the asymmetric case just the same. Specifically, Liskov and Wing's substitution principle [36] follows (precondition strengthening is reflected by the applicability constraints from $pre^A$ to $pre^C$, and all postconditions with respect to the outputs are equivalent). Data Refinement [15,25] follows similarly (Def. 1 characterizes that $\boldsymbol{R}$ is a forward simulation [37]). See Sect. 7 for more details.

### 3 Synthesis of Recursive Relational Invariants

In this section, we present the fundamentals of the approach to synthesize recursive relational invariants for systems over arrays and ADTs that we instantiate and illustrate on examples in the subsequent sections.

#### 3.1 Overview

Our approach is purely symbolic and fully automatic in both stages: generating a candidate relational invariant, and proving it correct (i.e., validating). The key insight is an analysis of the operations joint in the constraints of Def. 1. We follow a strategy of introducing recursion into the interpretation based on ADTs, and by aligning the base case to initialization and the recurrence conditions to joint operations. In particular, a relational invariant $\boldsymbol{R}$ that bridges an algebraic list $xs$

**Algorithm 1:** Automated synthesis of recursive relational invariants

**Input:** Objects $A = (as, Init^A, (Op_n^A)_{n \in N})$ and $C = (cs, Init^C, (Op_n^C)_{n \in N})$, where $as$, $cs$ are the state variables, and $xs$ is a list variable of $as$

**Output:** relational invariant $\boldsymbol{R}$ between $A$ and $C$

1 $\boldsymbol{R}(\texttt{nil}, cs) \leftarrow Init^A(as[xs := \texttt{nil}]) \wedge Init^C(cs)$;

2 $\phi_r \leftarrow true$;

3 let $y$ and $ys$ be fresh variables;

4 **while** *true* **do**

5 $\quad cs_r \leftarrow \textsc{Update}(Op_n^A, Op_n^C, as[xs := \texttt{cons}(y, ys)], cs)$ for some $n \in N$;

6 $\quad \phi_r \leftarrow \phi_r \wedge \textsc{Match}(Op_m^A, Op_m^C, as[xs := \texttt{cons}(y, ys)], cs, cs_r)$ for some $m \in N$;

7 $\quad \boldsymbol{R}(as[xs := \texttt{cons}(y, ys)], cs) \leftarrow \phi_r \wedge \boldsymbol{R}(as[xs := ys], cs_r)$;

8 $\quad$ **if** $\textsc{Validate}(\boldsymbol{R}, A, C)$ **then return** $\boldsymbol{R}$; and an array (with auxiliary variables, such as *index*) $cs$ is defined recursively over the structure of $xs$, which produces this general schema:

$$\boldsymbol{R}(xs, cs) = \begin{cases} \phi_b(cs) & \text{if } xs = \texttt{nil} \\ \exists\ cs_r.\ \phi_r(y, ys, cs, cs_r) \wedge \boldsymbol{R}(ys, cs_r) & \text{if } xs = \texttt{cons}(y, ys) \end{cases} \tag{1}$$

This schema has two placeholders for constraints, $\phi_b$ in the base case and $\phi_r$ in the recursive case, that may refer to the variables in scope (as indicated by their respective parameter lists). Moreover, we seek a Skolem function to eliminate the existentially-quantified state variable $cs_r$ in the recursive position. Intuitively the desired Skolem function captures the delta between two array states that corresponds to the delta between $xs$ and $ys$.

Alg. 1 gives our top-level synthesis procedure for interpretations of $\boldsymbol{R}$. It takes as input two objects, $A$ and $C$, where $as$ and $cs$ are tuples variables that represent their respective states. We refer to primed versions of these state variables to as $as'$ and $cs'$, assuming that all $as$, $cs$, $as'$, and $cs'$ are distinct. The algorithm works with algebraic lists specifically and thus $as$ is assumed to have such a component given by the state variable $xs$. We denote by $as[xs := e]$ the updated vector of variables such that $xs$ is replaced in $as$ by symbolic expression $e$.

The base case of the interpretation of $\boldsymbol{R}$ is straightforward (line 1): the algorithm uses a predicate $Init^C$ and a predicate $Init^A$ in which the $xs$ variable is instantiated to $\texttt{nil}$. The inductive case of the interpretation of $\boldsymbol{R}$ is trickier (line 7). Because several different operations that *produce* state, *consume* state, or *do nothing* with a state are possible (see Def. 2 later in the section), some of them might contribute to different parts of the interpretation being synthesized. In particular, methods Match and Update are responsible for generating a body of $\boldsymbol{R}$. They are instantiated differently for our two recipes in Sect. 4 (applicable for stacks and queues) and Sect. 5 (applicable for (multi)sets and maps).

The first method, Update, synthesizes an updated symbolic state $cs_r$, a tuple of symbolic expressions, to be used in the nested inductive call of $\boldsymbol{R}$. It can therefore be understood to compute a witness (or Skolem function) to existential quantifier in Eq. (1) as an expression of the remaining variables in scope, $y, ys, as, cs$. The second method, Match then collects constraints $\phi_r$ from suitable transitions w.r.t. this $cs_r$.

In a loop for each candidate interpretation of $\boldsymbol{R}$, our algorithm runs an automated SMT-based theorem prover [55] to validate it (line 8). The algorithm can iterate several times and converges after a successful theorem-prover run.

A noteworthy feature of our framework is that Update and Match should not necessarily be synchronized in pairs. Although $cs_r$ and the result of Match are going to be eventually combined and used in a single formula, the nondeterministic nature of our synthesis procedure suggests that the two ingredients may originate from potentially non-joint operations, thereby enlarging the search space of possible relational invariants.

#### 3.2 Classifying Operations

Our particular strategies for choosing ingredients for the inductive interpretation of $\boldsymbol{R}$ are based on the classification of the operations of the abstract object.

We define a partial ordering "$\preceq$" on ADT states that connects constructors discerned by the recurrence in $\boldsymbol{R}$ to the transitions of operations. With respect to this ordering, we can for example recognize operations that leave the ADT unchanged ("noops", which play a special role in Sect. 5), operations that "produce" constructors and thereby enlarge the internal state by additional elements and conversely operations that "consume" constructors. A natural choice for $\preceq$ is the reflexive closure of the subterm ordering, where $xs \preceq ys$ for lists specifies that $xs$ is a suffix of $ys$. In general, this ordering can be used to control the result of the synthesis for specific applications, and is a heuristic choice. A choice which works well for our examples is that $xs$ is a non-strict subsequence of $ys$.

The $\preceq$ ordering naturally extends to tuples of variables (and thus, states), and lets us classify operations into the following three kinds.

**Definition 2.** *Let Op be an operation of an abstract object. Then,*

$$\begin{aligned}
\textsc{isNo}(Op) &\stackrel{\text{def}}{=} \forall i, s, s', o \,.\, Op(i, s, s', o) \implies s = s' \\
\textsc{isProd}(Op) &\stackrel{\text{def}}{=} \forall i, s, s', o \,.\, Op(i, s, s', o) \implies s \preceq s' \wedge \neg\textsc{isNo}(Op) \\
\textsc{isConsm}(Op) &\stackrel{\text{def}}{=} \forall i, s, s', o \,.\, Op(i, s, s', o) \implies s' \preceq s \wedge \neg\textsc{isNo}(Op)
\end{aligned}$$

*Example 1.* The class of an operation can often be identified by a cheap syntactic check to recognize when `cons` is applied to a current state or a next state variable. In the upcoming stack example in Fig. 1, from $\mathtt{xs}' = \mathtt{cons}(\mathtt{in}, \mathtt{xs})$ we have that `push` is a producer operation, and from $\mathtt{cons}(\mathtt{out}, \mathtt{xs}') = \mathtt{xs}$ we classify `pop` as consumer operation. A `top` operation, not shown in Fig. 1, would be recognized as a noop (see also `hasElement` in the upcoming example in Fig. 3).

In the next two subsections, we introduce our particular strategies for the implementations of Update and Match of Alg. 1, in reference to Def. 2. Some operations fall into neither of the classes; or it may be hard to determine so if they do, given that Def. 2 is semantic; and different operations may contribute different ingredients for a correct definition of $\boldsymbol{R}$. To make use of as many operations as possible, we suggest strategies for all three classes of operations, to be able to synthesize a relational invariant in complex cases, even when complete information about the system is difficult to obtain.

## 4 Recipe 1: Linear Scan

We identify a class of problems that require *scanning* the arrays in implementations of stacks and queues *linearly.* A distinguishing feature in this class is the presence of a numeric variable in *cs* through which array cells are accessed (denoted *index* in the rest of the section). We first illustrate the synthesis process on the following example and then present the algorithmic details.

### 4.1 Motivating Example

Two realizations of a FIFO stack are shown in Fig. 1: one is based on linked lists, and another is based on arrays. They share a common interface of initialization and the two operations `push` and `pop`. For example, the encodings of `pop` of `ListStack` and `ArrStack` are respectively:

$$
\begin{aligned}
Op^{\mathsf{ListStack}}_{\mathsf{pop}}&(xs, xs', out) \\
&= (xs \neq \mathtt{nil} \wedge xs' = xs.\mathtt{tail} \wedge out = xs.\mathtt{head}) \\
&= (xs = \mathtt{cons}(out, xs')) \qquad\qquad \text{(after simplification)} \\
Op^{\mathsf{ArrStack}}_{\mathsf{pop}}&(a, n, a', n', out) \\
&= (n > 0 \wedge a' = a \wedge n = n - 1 \wedge out = a[n'])
\end{aligned}
$$

where $xs \neq \mathtt{nil}$ and $n > 0$ are the preconditions, and *out* captures the return value. As an illustration, formula $Op^{\mathsf{ListStack}}_{\mathsf{pop}}(s, \_, 7)$ holds for all states $s$ in which `pop` terminates and returns 7 (by convention we use _ to denote terms that are irrelevant in a particular context). Note also that in the implementation of `ArrStack`, the popped value is not erased from the array – in order for $a[n]$ to be considered in the future, it has to be rewritten by some `push` operator. In general, the array always contains infinitely many unknown values outside the range of cells $a[0], \ldots, a[n-1]$ which are never accessed.

A possible relational invariant $\boldsymbol{R}(xs, n, a)$ bridging `ListStack` and `ArrStack` is defined as follows:

$$
\boldsymbol{R}(xs, n, a) = \begin{cases} n = 0 & \text{if } xs = \mathtt{nil} \\ n > 0 \wedge y = a[n-1] \wedge \boldsymbol{R}(ys, n-1, a) & \text{if } xs = \mathtt{cons}(y, ys) \end{cases} \qquad (2)
$$

Intuitively, this $\boldsymbol{R}$ captures that a list *xs* has the same content as the portion of an array $a$ between indexes 0 (including) and $n$ (excluding). When *xs* is empty, then the portion of $a$ should be empty too, thus $n = 0$. Otherwise, *xs* is created by `cons`-ing some other list *ys* and an element $y$ then (1) $n$ should be strictly positive, and (2) $y$ should belong to the designated portion of $a$.

```
class ListStack:
  def init():
    xs = nil def push(in):
    xs = cons(in, xs)

def pop():
    assert xs != nil
    out = xs.head
    xs = xs.tail
    return out
```

```
class ArrStack:
  def init():
    n = 0
    a = [...]

def push(in):
    a[n] = in
    n = n + 1

def pop():
    assert n > 0
    n = n - 1
    return a[n]
```

**Fig. 1:** Two implementations of a FIFO stack.

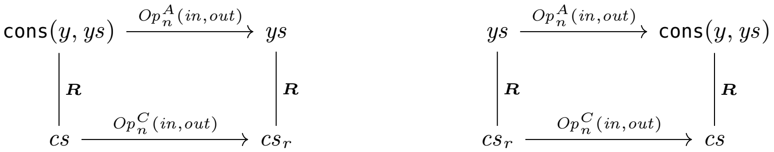

**Fig. 2:** Transitions of consumer operations (left) and producer operations (right) used to instantiate Eq. (1).

The schema in Sect. 3.1 has two placeholders for constraints, $\phi_b$ in the base case and $\phi_r$ in the recursive case, that may refer to the variables in scope (as indicated by their respective parameter lists). Moreover, we seek a state $cs_r$ in the recursive position. Placeholder $\phi_b$ is instantiated by constraints from the initialization operations, such as $n = 0$ from `ArrStack`. This alignment of base case and initialization is not just a coincidence: many data structures start initially empty and are gradually populated by calling operations (e.g., collections).

The purpose of $\phi_r$ in the recursive case of Eq. (1) is twofold. First, it connects a portion of the ADT state (specifically $y$) to the array state $cs$, in the example via $a[n-1] = y$, and it determines a suitable array state $cs_r$ as an argument of the recursive occurrence of $\boldsymbol{R}$. For instance, we take $n - 1$ for the recursive call but leave $a$ unchanged. This is motivated by the observation that a state where $xs = \texttt{cons}(y, ys)$ for some $y, ys$ is *consumed* by `pop`. Using this information, the recurrence of $\boldsymbol{R}$ must align with the corresponding array transitions, too, as shown in Fig. 2 on the left. The constraint $n > 0$ is the precondition of the array operation, whereas $y = a[n-1]$ follows from comparing the outputs. As shown in Fig. 2 on the right, we can dually base the recurrence on `push`, which *produces* a `cons`, i.e., a transition from $ys$ to $xs = \texttt{cons}(y, ys)$ for some $y$. In this case, both transitions need to be viewed *in reverse* such that the respective successor states of `push` now match the left side $\boldsymbol{R}(xs, cs)$ of the schema. Then, the assignment `n = n + 1` can be rewritten to yield the equation $n_r = n - 1$.

**Algorithm 2:** UPDATE (recipe 1)

**Input:** Operations $Op^A$ and $Op^C$,
$as[xs := \mathtt{cons}(y, ys)]$ the shape of the state of $A$,
$cs$ the state variables of $C$, assuming $cs = (\_, index, a)$ where $index$ and $a$ are variables of integer and array types, resp.
**Output:** Updated arguments $cs_r$

```
1 if isProd(Op^A) then
2     let cs_r = (_, index', a') be s.t. ∀in, ∃out . Op^C(in, cs_r, cs, out);
3     return (_, index', a);
4 if isConsm(Op^A) then
5     let cs_r = (_, index', a') be s.t. ∀in, ∃out . Op^C(in, cs, cs_r, out);
6     return (_, index', a);
```

**Algorithm 3:** MATCH (recipe 1)

**Input:** Operations $Op^A$ and $Op^C$,
$as[xs := \mathtt{cons}(y, ys)]$ the shape of the state of $A$,
$cs$ the state variables of $C$,
$cs_r$ the updated state of $C$, assuming $cs_r = (\_, index', a)$ where $index'$ and $a$ are variables of integer and array types, resp.
**Output:** Formula $\phi_r$

```
1 if isProd(Op^A) then
2     inv ← GetLoopInvariant(index', Op^C);
3     return inv ∧ ¬Init^C(cs) ∧ y = a[index'];
4 if isConsm(Op^A) then
5     return pre_n^A ∧ pre_n^C ∧ y = a[index'];
6 return true;
```

To make this intuition practical, our approach suggests a particular strategy for picking operations to take constraints from, recognizing consumers and producers more generally, and validating the guessed relational invariants using induction and lemmas.

### 4.2 Algorithm Description

Alg. 2 and Alg. 3 show the implementations of UPDATE and MATCH, respectively, that suit stacks and queues. Recall that these algorithms are called from Alg. 1 and take as input pairs of nondeterministically chosen joint operations of $A$ and $C$; state variables $cs$ of $C$; current version of state variables $cs_r$ to be used in the recursive call of $\boldsymbol{R}$; and fresh variables $y$ and $ys$ introduced in Alg. 1 to define the inductive rule of $\boldsymbol{R}$. Outputs of UPDATE and MATCH are respectively an updated tuple of variables $cs_r$ and a subformula $\psi$ to be conjoined with the inductive definition of $\boldsymbol{R}$.

If the producing operator is picked (line 1 of Alg. 2), then we have to find a term $index'$, such that it would be transitioned by $Op^C$ to $index$. In particular, after assigning a new value to an array cell, *index* is monotonically updated (i.e., incremented like in the example in Fig. 1, or decremented). Thus, to access the array cell containing a new value using an updated value of *index*, we have to invert the arithmetic operation and obtain $index - 1$ (for Fig. 1) or $index + 1$ (in the case of decrementation). Technically, in Alg. 2, it is realized by taking the *index* variable from $cs$, through which cells of the array can be observed (e.g., $n$ in example in Fig. 1) and finding such a term $index'$, that would be transitioned by $Op^C$ to *index*. Thus, the resulting $cs_r$ is composed from the same ingredients as $cs$ where $index'$ replaces *index*.

If the consuming operation is picked (line 4), then we proceed in the reverse direction and find $index'$ that is a result of transitioning of *index* through $Op^C$.

Alg. 3 for this recipe relies on the output of Alg. 2. Interestingly, it is supported even if $cs_r$ is computed using the producer, but $\psi$ in Alg. 3 is computed using the consumer. Our particular strategy for the consumers in this recipe is 1) to use the precondition for $Op^C$, and 2) to bridge the outputs of $Op^A$ and $Op^C$ via an equality. Alternatively, the inference via producer in line 1, in comparison, misses important constraint in the example, as the precondition of `push` is trivial. Such a situation can be mitigated by the discovery of a loop invariant (line 2) over *index*, i.e., usually just using Linear Integer Arithmetic (LIA), adding it, and blocking the initial state (to distinguish from the base case of the definition of $\boldsymbol{R}$) in the inductive case of the interpretation of $R$ being synthesized. Loop invariants are generated as follows as interpretations of predicate *inv* satisfying the following two implications:

$$\begin{aligned} Init^C(cs) &\implies inv(cs) \\ inv(cs) \wedge \left( \bigvee_{n \in N} Op^C_n(in, cs, cs', out) \right) &\implies inv(cs') \end{aligned}$$

Note that these CHCs (over LIA) can be solved by numerous existing approaches. Without a query, ideally *the strongest* loop invariant is desirable; however in practice it suffices to apply lightweight techniques based on forward-propagation of initial states using quantifier elimination, followed by its inductive subset computation [20]. This often finds an *adequately-strong* invariant.

*Example 2.* Recall the stack example in Fig. 1. Let the $index'$ term be computed by Alg. 2 via inverting the increment operation in `push`. Thus, it is used as an argument of the nested call to $\boldsymbol{R}$ in the inductive case of the definition of $\boldsymbol{R}$. By construction, the $a[index']$ cell contains a value of `in`, i.e., the argument of `push`. At the same time, `in` is the argument of `cons` in $Op^A$ representing `push`, which lets us bridge the array and ADT in the proof. To allow this, Alg. 3 takes argument $y$ of `cons` from the inductive definition of $\boldsymbol{R}$, and equates it with $a[index']$, producing $y = a[n-1]$. Combining it all together, we get the final solution, as shown in (2).

```
class ListSet:
  def init():
    xs = nil def hasElement(in):
    return contains(xs, in)

def insert(in):
    xs = cons(in, xs)

def erase(in):
    xs = removeall(xs, in)
```

```
class ArraySet:
  def init():
    a = [false, false, ...]

def hasElement(in):
    return a[in]

def insert(in):
    a[in] = true def erase(in):
    a[in] = false
```

**Fig. 3:** Two implementations of a set, where the list is not necessarily duplicate-free.

### 5 Recipe 2: Noop-based synthesis

In this subsection we present a recipe that suits sets, multisets, and maps, that are in some sense *non-linear*. That is, data structures do not maintain any *index* variable, which is usually used to access elements. Instead, arrays are viewed as maps, and the corresponding ADTs are equipped with recursive functions that traverse the data structure over and over again for each input. Oftentimes, these objects have noop operations, and our synthesis procedure makes use of them.

#### 5.1 Motivating Example

Fig. 3 shows two implementations of a set. The list-based implementation stores elements in the order of their `insert`-ions. The elements are not removed unless `erase` is called explicitly. Thus, duplicate entries of the same elements are allowed. The implementation uses the recursive `contains` and `removeall` functions that both traverse the list and search for a specific element:

$$\texttt{contains}(xs, a) = \begin{cases} \mathit{false} & \text{if } xs = \texttt{nil} \\ (a = y) \vee \texttt{contains}(ys, a), & \text{if } xs = \texttt{cons}(y, ys) \end{cases}$$

$$\texttt{removeall}(xs, a) = \begin{cases} \texttt{nil} & \text{if } xs = \texttt{nil} \\ \mathit{ite}(a = y, \texttt{removeall}(ys, a), & \\ \texttt{cons}(y, \texttt{removeall}(ys, a))) & \text{if } xs = \texttt{cons}(y, ys) \end{cases}$$

The array-based implementation handles a map $a$ from elements to Booleans. Initially, all cells in $a$ are false. Inserting and removing an element is implemented by storing `true` and `false` to the corresponding cell respectively. The difficulty here is to support the shown implementation of `insert` and `erase` in Fig. 3, as well as possible variants that e.g., eagerly prune duplicate entries in the list-based implementation (see Sect. 6).

The expected output of our synthesis procedure is as follows:

$$\boldsymbol{R}(xs, a) = \begin{cases} \forall z.\ \neg a[z] & \text{if } xs = \texttt{nil} \\ a[y] \wedge \boldsymbol{R}(ys, a[y := \texttt{contains}(ys, x)]), & \text{if } xs = \texttt{cons}(y, ys) \end{cases} \qquad (3)$$

**Algorithm 4:** UPDATE (recipe 2)

**Input:** Operations $Op^A$ and $Op^C$ such that $\textsc{isNo}(Op^A)$ holds,
$as[xs := \texttt{cons}(y, ys)]$ the shape of the state of $A$,
$cs$ the state variables of $C$
**Output:** Updated arguments $cs_r$

let $cs'$ be fresh variables;
$\phi \leftarrow Op^A(y, as[xs := ys], as[xs := ys], out) \wedge Op^C(y, cs', \_, out)$;
$\psi \leftarrow \forall z\,.\, z \neq y \implies \exists out'\,.\, Op^C(z, cs, \_, out') \wedge Op^C(z, cs', \_, out')$;
assume $\mathrm{QE}(\exists out\,.\, \phi \wedge \psi)$ simplifies to $(cs' = cs_r)$;
**return** $cs_r$;

**Algorithm 5:** MATCH (recipe 2)

**Input:** Operations $Op^A$ and $Op^C$ such that $\textsc{isNo}(Op^A)$ holds,
$as[xs := \texttt{cons}(y, ys)]$ the shape of the state of $A$, denoted $as_0$ below,
$cs$ the state variables of $C$,
$cs_r$ the updated state of $C$
**Output:** Formula $\phi_r$

$\phi \leftarrow Op^A(y, as_0, as_0, out) \wedge Op^C(y, cs, cs_r, out)$;
**return** $\textsc{simplify}(\mathrm{QE}(\exists out\,.\, \phi))$;

#### 5.2 Algorithm details

Alg. 4 and Alg. 5 show the implementations of UPDATE and MATCH, respectively, for this recipe. The arguments $cs_r$ of the nested call to $\boldsymbol{R}$ in the inductive case of the definition of $\boldsymbol{R}$ are computed in Alg. 4 using the symbolic encoding of *noop*. In the set example, *noop* is the `hasElement` operation, which allows observing the status of the internal state and does not modify it. We furthermore assume that the input of $Op_n$ coincides with the type of elements stored in the list, i.e., it is meaningful to call $Op_n(y, \cdots)$ with the list head $y$ from the recursive case of (1) where $xs = \texttt{cons}(y, ys)$.

The key idea behind Alg. 4 is to make necessary adjustments to $cs$ to construct $cs_r$ that mirror any changes that can be observed via $Op^A$ when transitioning from list $xs$ to $ys$ in (1). This update is determined in terms of an auxiliary variables $cs'$ that are constrained to satisfy certain input/output pairs for the corresponding $Op^C$, by case analysis whether the input is this particular $y$ that is removed by the recurrence. The primary intention is to reassign $a[y]$ appropriately. We do this by collecting constraints $\phi$ such that the output observed for $Op^C$ for $y$ and $cs'$ matches that of the corresponding $Op^A$ on the smaller state with $ys$. This is also the key difference to Sect. 4, where we heuristically keep $a$ unchanged in the recursive call in (1). The outputs for all other inputs $z$, however, are enforced to be unchanged w.r.t. the original $cs$, which is expressed by the constraint $\psi$. We then eliminate the quantifier for $out$ (which is straightforward as the operations are deterministic) and rewrite the formula to closed expressions $cs_r$ for variables $cs'$ as result.

*Example 3.* Specifically for the example in Sect. 5.1, the algorithm proceeds by symbolic execution of `hasElement`, yielding formulas the following constituents:

$$\begin{aligned} Op^A &= (out = \mathtt{contains}(ys, y)) \\ Op^C &= (out = a[y]) \\ \phi &= (out = \mathtt{contains}(ys, y) \land out = a'[y]) \\ \psi &= (\forall z \,.\, y \neq z \implies \exists out' \,.\, out' = a'[z] \land out' = a[z]) \end{aligned}$$

The result $\exists out \,.\, \phi \land \psi$ of Alg. 4 is now solved for $a'$. The only free variables refer to the states of the systems. Bound variables $out$ and $out'$ can be eliminated by merging equalities over $out$ and $out'$:

$$a'[y] = \mathtt{contains}(ys, y) \land (\forall z \,.\, y \neq z \implies a'[z] = a[z])$$

The first conjunct therefore provides the update for $a'[y]$, whereas the second conjunct of $\phi$ states that $a'[z]$ should *not* be changed at indices other than $y$. After applying the axioms over the theory of arrays we get as result the following equality, which pattern matches the expected shape in line 4:

$$\mathrm{QE}(\exists out \,.\, \phi) \iff (a' = a[x \coloneqq \mathtt{contains}(ys, x)])$$

This transformation requires to "reverse-apply" the axiom of extensionality, i.e., switch from the pointwise comparison of $a$ and $a'$ to an equality between the entire arrays. Note that while in general quantifier elimination is difficult, our current implementation has a limited, but often sufficient, support that can be extended by supplying rules to the underlying SMT-based theorem prover.

While $Op^A$ Alg. 4 predict *future* outputs of $Op^A$ for input $y$, Alg. 5 executes $Op^A$ on the state where $xs = \mathtt{cons}(y, ys)$ to obtain the *current* output of $Op^A$ for the same $y$. The generated constraint simply expresses that the output of $Op^C$ has to match. For `hasElement` we obtain the following formula:

$$\exists out \,.\, (\mathtt{contains}(\mathtt{cons}(y, ys), y) = out) \land (a[y] = out)$$

Unfolding the definition of `contains` and simplification produces $true = a[x]$, which is then used as the "*body*" of the inductive case of $\boldsymbol{R}$ in (3).

### 6 Evaluation

We have implemented the approach in a prototype CHC solver called AdtChc[3], relying on AdtInd [55] as an inductive prover, which in turn uses the Z3 [40] SMT solver to quickly perform the satisfiability checks over uninterpreted functions and linear arithmetic that are needed at various solving stages. AdtChc automatically determines the appropriate synthesis recipe through analyzing the

[3] The tool and benchmarks are available at https://github.com/grigoryfedyukovich/aeval/tree/adt-chc.

syntax of the program (i.e., presence of index variables) and is able to successfully find relational invariants and prove them valid for all considered benchmarks.

We have evaluated the approach from Sect. 3 on different realizations of text-book data structures. The evaluation aims at answering two questions. Is the approach effective in the first place to discover suitable relational invariants, and how well can the necessary induction proofs be automated? The latter is relevant since Alg. 1 crucially depends on VALIDATE in its refinement loop.

All our benchmarks require recursive invariants. They fall into two categories. First, stacks and queues from Sect. 4 (with variations that store values only to even indexes of the array) are solved based on linear scan. Second, sets, multisets, and maps, (that differ in whether, e.g., duplicate elements are stored in the respective lists) are solved with the approach in Sect. 5. We include such variations to reflect different trade-offs when designing specifications, and to demonstrate that our technique is reasonably flexible. The only user-provided lemma was required for the multiset benchmark (marked * in Table 1): $\forall\, a, xs.\ \texttt{num}(a, xs) = 0 \implies \texttt{remove}(a, xs) = xs$.

The results from the evaluation[4] of both groups of benchmarks (resp., recipes used) are shown in Table 1. The choice which recipe to use was made by the tool itself at synthesis time. Total time (in seconds wall-clock) is entirely dominated by proof search in ADTIND, and includes the time for SMT queries. We remark that the time to synthesize the relational invariant is negligible in comparison to the proof time (and the proof time is often proportional to the number of internal SMT calls).

**Table 1:** Invariant synthesis timings.

| Benchmark | Variant | Time (s) |
|---|---|---:|
| Stack | Fig. 1 | 2.81 |
| Stack | even cells | 2.79 |
| Queue | ordinary | 40.61 |
| Queue | even cells | 42.18 |
| Set | Fig. 3 | 2.12 |
| Set | no duplicates | 19.24 |
| Multiset* | with `remove` | 32.62 |
| Multiset | with `clear` | 3.59 |
| Map | duplicates | 1.95 |
| Map | no duplicates | 5.83 |

Most proofs are found using the default proof strategy (the same for every benchmark) within 20s. This is caused by the large proof search space created by a combination of array simplification and forward rewriting. We have also tested our tool of buggy implementations, e.g., in which the consumer operations are correct (and can be used for correct guesses of relational invariants), but producers are not. Expectedly, the tool is unable to synthesize a relational invariant for the whole systems in these cases.

We have already presented the relational invariants found for the stack (2), for the stack variant that stores to even array indices only, counter $n$ is decreased by 2 instead of 1 in the recursive call as expected. Relational invariant $\boldsymbol{R}(xs, m, n, a)$ for the queue benchmarks keeps two indices into the array $a$, depending on the variant, the first element of the list $xs$ is found at $a[m]$ or $a[n]$

[4] The evaluation was conducted on MacBook Pro, Processor: 2 GHz Intel Core i5, Memory: 8 GB 1867 MHz LPDDR3, MacOS v10.14.6.

and the recursion either increases $m$ or decreases $n$. The relational invariants for the multiset and map examples are analogous. All necessary lemmas are automatically discovered and proved by AdtInd, as an example for the set benchmarks: $\forall\ xs, s, x.\ \boldsymbol{R}(xs, s) \implies \texttt{contains}(x, xs) = s[x]$.

### 7 Related Work

Although there exist automated techniques to synthesize relational invariants, nothing was proposed to deal simultaneously with ADTs and arrays. Conceptually, our approach is related to SimAbs, an SMT-based algorithm to simulation synthesis [18]. SimAbs exploits a space of possible simulations and (dis-)proves them using an off-the-shelf decision procedure. Guesses for simulation relation are obtained also from the source code, by matching variables from two programs. Alternatively, simulation relations can be inferred from test runs [49] or through translation validation [41]. Our approach allows dealing with objects (not just imperative code) and contributes several novel strategies for guessing and proving non-trivial simulation relations.

Discovery of invariants to relate the behaviors of two programs or other ways of establishing program equivalence is an active research area [5,14,22,23,39,44,51]. These approaches typically reduce the relational verification problem to a safety verification problem and rely on the existing tools—often, solvers for constrained Horn clauses (CHC). Currently, since ADTs and arrays are challenging for the underlying solvers, the applicability of the approaches to our tasks are also limited. There are decision procedures for abstraction of ADTs to lists, sets, and multisets [52], however, these apply to certain predefined abstractions only.

Our approach can be seen as an application of Syntax-Guided Synthesis (SyGuS) [2]. Strategies dependent on types of benchmarks essentially represent sets of syntactic templates filled iteratively and checked using an SMT solver. SyGuS is successfully used also in CHC solving [19,21] and in lemma synthesis [46,47,55]. There are only a few approaches [21,28,31,55] that apply SyGuS to synthesize formulas over ADTs or arrays/quantifier. Data-driven approaches are complementary to such syntax-based approaches, e.g., [38]. Neither deals with arrays, quantifiers, and ADTs at the same time.

Unno et al. [53] support recursive predicates, by taking the least solution of initialization and consecution as the definition of $\boldsymbol{R}$, however, this may lead to rather cumbersome inductive cases (e.g., for `pop` in the stack). We avoid the problem by basing the recurrence scheme on the data structure, and infer constraints that are well aligned to that scheme from the operations. Jennisys [34] tackles the related problem of generating recursive implementations from an abstract model, where the simulation relation is given.

More generally, the problem addressed in this work relates to the idea of step-wise refinement, originally conceived by [16] and [54] as a guideline to organize software development and later studied extensively in a formal setting for rigorous assessment of functional correctness (e.g., [1,4,15,25,29,33,36]). The standard proof technique relies on simulation relations [37] that couple the two state spaces, which is directly reflected in the CHC system of Def. 1.

Many methods and tools support development using formal refinement [1,4, 8,17,26,29,33,45]. Large-scale verification projects that are based on refinement include `seL4` [30], `FSCQ` [10], `Flashix` [48], and `CompCert` [35], with high human effort involved. Correct-by-construction correspondence between low-level code and high-level data types helps to some extent in, e.g., [13] and Cogent [3]. Recent work on "push-button" verification includes a verified `TLS` library [12], `AWS C Common` library [11], file system [50], a hyperkernel [42], network functions [56], where the high degree of proof automation is in part achieved by statically bounding the state space of the systems. The latter work [56] specifically notes how non-experts can formulate high-level correctness requirements (their specifications are written in Python), as evidence that refinement-based approaches may ultimately overcome the "specification bottleneck" [6,43].

### 8 Conclusion and Outlook

We have demonstrated an approach that can fully automatically synthesize and prove relational invariants over recursive data types and arrays. The approach is based on introducing quantifiers and recursion into the definition of such relations in a systematic way, and by instantiating this schema with constraints from joint transitions of the two systems. A somewhat surprising insight was that it is useful to view such transitions both forward and in reverse, leading to the classification into producers and consumers as a guideline for the search.

We have presented a general synthesis algorithm and two concrete instantiations for different data structures of different sorts. The approach is fully automatic in guessing a relation and proving it correct. It relies on the recently developed CHC solver called AdtChc which in turn is based on an SMT-based theorem prover AdtInd featuring a support for arrays, quantifiers and structural induction. The approach is modular and can be extended by further synthesis strategies in the future. In particular, since based on CHC techniques, it can be integrated with other existing CHC solvers tailored to non-ADT reasoning, and can be used in large-scale verification frameworks such as [24] that reduce the safety verification to CHC tasks.

Many more interesting benchmarks lend themselves for further investigation: positional insertion and removal of lists, amortized data structures, benchmarks based on trees or nested arrays, and ultimately some real-world software systems. With a growing search space, it becomes more important to quickly recognize incorrect simulation relations, e.g., by evaluation-based counter-examples (cf. [31]), to prevent costly proof attempts. Similarly, incorporating external tools for invariant generation is another topic for future work.
